# The effect of magnetic field on the impurity binding energy of shallow donor impurities in a Ga_1−*x*_In_*x*_N_*y*_As_1−*y*_/GaAs quantum well

**DOI:** 10.1186/1556-276X-7-586

**Published:** 2012-10-24

**Authors:** Unal Yesilgul, Fatih Ungan, Serpil Şakiroğlu, Carlos Duque, Miguel Mora-Ramos, Esin Kasapoglu, Huseyin Sari, Ismail Sökmen

**Affiliations:** 1 Physics Department, Cumhuriyet University, Sivas, 58140, Turkey; 2 Physics Department, Dokuz Eylül University, Izmir, 35140, Turkey; 3 Instituto de Fisica, Universidad de Antioquia, Medellin, AA, 1226, Colombia; 4 Faculty of Sciences, Morelos State University, Cuernavaca, CP, 62209, Mexico

**Keywords:** Impurities, Quantum well, Dilute nitride

## Abstract

Using a variational approach, we have investigated the effects of the magnetic field, the impurity position, and the nitrogen and indium concentrations on impurity binding energy in a Ga_1−*x*_In_*x*_N_*y*_As_1−*y*_/GaAs quantum well. Our calculations have revealed the dependence of impurity binding on the applied magnetic field, the impurity position, and the nitrogen and indium concentrations.

## Review

### Background

Over the past decade, the GaInNAs-based quantum-well structures have emerged as a subject of considerable theoretical and experimental research interest due to their very unique physical properties and due to a wide range of possible device applications. GaInNAs exhibits interesting new properties and differs considerably from the conventional III to V alloys. Significant changes occur in the electronic band structure compare with GaInAs with incorporation of small amounts of nitrogen into GaInAs. These include a large redshift of the bandgap, a highly nonlinear pressure dependence of the bandgap, an increase in the electron effective mass, and the N-induced formation of new bands [1-10]. This new material has received considerable attention due to the growing interest in its basic physical properties. Shan et al. showed that interaction between the conduction band and narrow resonant band formed by nitrogen states in GaInNAs alloys leads to a splitting of conduction band into sub-bands and a reduction of the fundamental bandgap [11]. Fan et al. have investigated the electronic structures of strained Ga_1−*x*_In_*x*_N_*y*_As_1−*y*_/GaAs quantum wells [12]. Hetterrich et al. investigated the electronic states in strained Ga_0.62_In_0.38_N_0.015_As_0.985_/GaAs multiple quantum-well structures [13]. Pan et al. have investigated the optical transitions in Ga_1−*x*_In_*x*_N_*y*_As_1−*y*_/GaAs single and multiple quantum wells using photovoltaic measurements at room temperature [14]. Several studies have been done on detailed optical characterization of Ga_1−*x*_In_*x*_N_*y*_As_1−*y*_. These papers include the temperature dependence of photoluminescence, absorption spectrum, and low-temperature photoluminescence [15-19].

There are many studies associated with the hydrogenic binding of an electron to a donor impurity which is confined within low-dimensional heterostructures [20-25]. The understanding of the electronic and optical properties of impurities in such systems is important because the optical and transport properties of devices made from these materials are strongly affected by the presence of shallow impurities. Also, it is well known that a magnetic field considerably affects the optical and electronic properties of semiconductors. Thus, the effects of magnetic field on the impurity binding energy are a very important problem [26-28]. However, up to now, to the best of our knowledge, no theoretical studies have been focused on impurity binding energies in single GaInNAs/GaAs quantum well (QW) under the magnetic field.

In this paper, using a variational technique within the effective mass approximation, we have investigated the effects of the magnetic field, the impurity position, and the nitrogen (N) and indium (In) concentrations on impurity binding energy in a Ga_1 − *x*_In_*x*_N_*y*_As_1 − *y*_/GaAs QW.

#### Theoretical overview

The growth axis is defined to be along the *z*-axis and takes the magnetic field to be applied to the *z*-axis, i.e., **B** = (0, 0, *B*). We choose a vector potential **A** in written form $\text{A=}\frac{1}{2}\left(\frac{-B_{y}}{2},\frac{B_{x}}{2},0\right)$ to describe the applied magnetic field. Within the framework of the effective mass approximation, the Hamiltonian of a hydrogenic donor impurity in a Ga_1 − *x*_In_*x*_N_*y*_As_1 − *y*_/GaAs quantum well in the presence of magnetic fields can be written as follows:

(1) $$H=\frac{1}{2m^{*}}{\left[p_{e}+\frac{e}{c}A\left(r_{e}\right)\right]}^{2}+V\left(z\right)-\frac{e^{2}}{\varepsilon_{o}{\left|r_{e}-r_{i}\right|}^{\text{,}}}$$

where m^*^ is the effective mass, *e* is the elementary charge, **p**_e_ is the momentum of the electron, *ε*_*o*_ is the dielectric constant of the system, **r**_*e*_ and **r**_*i*_ are the electron and impurity atom positions, and *V*(*z*) is the confinement potential of the electron in the *z*-direction. Using cylindrical coordinates (*x* = *ρ* cos *ϕ*, *y* = *ρ* cos *ϕ z* = *z*) , we obtain the Hamiltonian as follows:

(2) $$\begin{array}{l} H=-\frac{\hbar^{2}}{2m^{*}}\left(\frac{\partial^{2}}{\partial \rho^{2}}+\frac{1}{\rho }\frac{\partial }{\partial \rho }+\frac{1}{\rho^{2}}\frac{\partial^{2}}{\partial \phi^{2}}\right)-\frac{\hbar^{2}}{2m^{*}}\frac{\partial^{2}}{\partial z^{2}}\\ \;+\frac{e^{2}B^{2}}{2m^{*}c^{2}}\rho^{2}+V\left(z\right)-\frac{e^{2}}{\varepsilon_{o}\sqrt{\rho^{2}+{\left(z-z_{i}\right)}^{2}}}\text{,} \end{array}$$

where the term $\rho =\sqrt{{\left(x_{e}-x_{i}\right)}^{2}+{\left(y_{e}-y_{i}\right)}^{2}}$ is the distance between the electron and impurity in the (*x* and *y*) plane. The location of the hydrogenic donor in the structure is given as (0, 0, *z*_*i*_). The electron confining potential, *V*(*z*), is taken as follows:

(3) $$V\left(z\right)=\{\begin{array}{cc} 0 & \left|z\right|\leq L/2\\ V_{0} & \text{elsewhere} \end{array}\text{,}$$

with *L* as the well width, and *V*_0_ is the conduction band offset which is taken to be 80% of the total discontinuity between the bandgap of GaAs and Ga_1 − *x*_In_*x*_N_*y*_As_1 − *y*_ grown on GaAs [13].

The bandgap energy and electron effective mass of Ga_1 − *x*_In_*x*_N_*y*_As_1 − *y*_/GaAs is calculated using the band anti-crossing model (BAC). The electron effective mass of Ga_1 − *x*_In_*x*_N_*y*_As_1 − *y*_/GaAs as predicted by the BAC model is given by the following [29,30]:

(4) $$\begin{array}{l} m^{*}\left(Ga_{1-x}{\text{In}}_{x}N_{y}{\text{As}}_{1-y}\right)=2m^{*}\left({\text{In}}_{x}{\text{Ga}}_{1-x}\text{As}\right)/\\ \;\left(1-\frac{E_{C}-E_{N}}{\sqrt{{\left(E_{C}-E_{N}\right)}^{2}+4V_{\mathrm{NC}}^{2}y}}\right)\text{.} \end{array}$$

The *E* in the BAC model is taken to be the fundamental bandgap energy (*E*_*G*_) for Ga_1 − *x*_In_*x*_N_*y*_As_1 − *y*_:

(5) $$E=\frac{1}{2}\left[\left(E_{N}+E_{C}\right)-\sqrt{{\left(E_{N}-E_{C}\right)}^{2}+4V_{\mathrm{NC}}^{2}y}\right]\text{,}$$

(6) $$E_{C}=E_{C0}-1.55y$$

(7) $$E_{N}=1.65\left(1-x\right)+1.44x-0.38x\left(1-x\right)$$

(8) $$V_{\mathrm{NC}}=2.7\sqrt{y\text{,}}$$

where *y* is the N composition in Ga_1 − *x*_In_*x*_N_*y*_As_1 − *y*_; *E*_C0_ is the energy in the absence of N; *E*_*C*_, *E*_*N*_, and *V*_*NC*_ are the bandgap energies of InGaAs at Г point, the energy of the isolated N level in the InGaAs host material, and the coefficient describing the coupling strength between *E*_*N*_ and the InGaAs conduction band, respectively. The band structure parameters used in this study are listed in Table 1[31-36].

**Table 1:** Parameters of the binary compounds used for the calculation

**Material**	**GaAs**^**a**^	**InAs**^**a**^	**GaN**^**b**^	**InN**^**b**^
Electron effective mass m^*^(*m*_0_)	0.067	0.026	0.15	0.14
Dielectric constant	12.53	14.55	10.69^c^	7.46^d^
Energy gap *E*_g_ (eV)	1.420	0.417	3.299	1.94

^a^Vurgaftman et al. [33], ^b^Vurgaftman and Meyer [34], ^c^Chow et al. [35], and ^d^Gavrilenko and Wu [36].

Using the variational method, it is possible to associate a trial wave function, which is an approximated eigenfunction of the Hamiltonian described in Equation 2. The trial wave function is given by the following:

(9) $$\text{Ψ}\left(r\right)=\text{ψ}\left(z\right)\phi (\rho \text{,λ),}$$

where λ is the variational parameter, *ψ*(*z*) is the wave function of the donor electron which is exactly obtained from the Schrödinger equation in the *z*-direction without the impurity, and *ϕ*(ρ,λ) is the wave function in the (*x* and *y*) plane, and it is given by the following:

(10) $$\phi \left(\rho ,\text{λ}\right)=\frac{1}{\text{λ}}{\left(\frac{2}{\pi }\right)}^{\frac{1}{2}}Exp\left[-\rho /\text{λ}\right]\text{.}$$

The ground state impurity energy is evaluated by minimizing the expectation value of the Hamiltonian in Equation 2 with respect to λ. The ground state donor binding energy is calculated using the following equation:

(11) $$E_{B}=E_{z}-\underset{\text{λ}}{\text{min}}\langle \text{Ψ}\left|H\right|\text{Ψ}\rangle \text{,}$$

where *E*_*z*_ is the confinement ground state energy of the electron.

## Results and discussion

In this paper, we have theoretically investigated the effects of the magnetic field, the impurity position, and the nitrogen and indium concentrations on impurity binding energy in a Ga_1 − *x*_In_*x*_N_*y*_As_1 − *y*_/GaAs QW.

**Figure 1:**
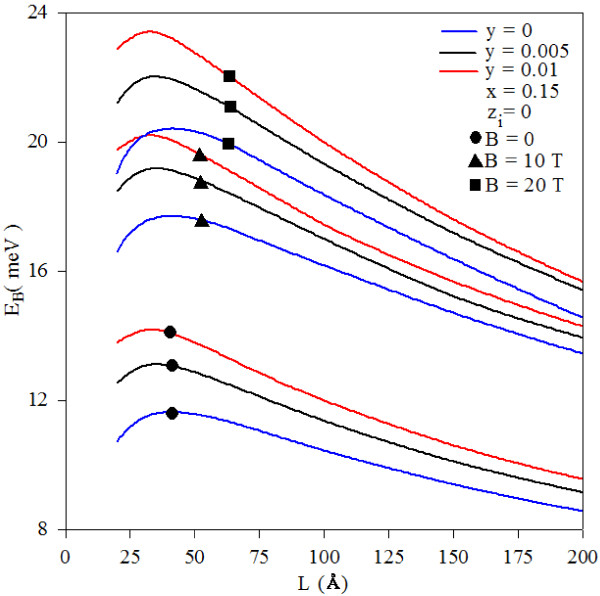
**Variation of impurity binding energy as function of well width**: **magnetic**-**field values and nitrogen concentrations.** This is for some values of the magnetic field (*B* = 0, 10, and 20 T) and nitrogen concentrations (*y* = 0, 0.005, and 0.01).

The variation of the impurity binding energy as a function of the well width for different values of the magnetic field and for two different nitrogen concentrations (*y* = 0, *y* = 0.005, and *y* = 0.01) is given in Figure 1. As seen in this figure, when the magnetic field increases, the binding energy also increases. Magnetic field strength gives an additional parabolic magnetic confinement term. Under this additional magnetic confinement, the probability of the electron and impurity atom being on the same plane increases, and therefore, the binding energy increases. In this figure, one sees that when the well width increases, the impurity binding energies increase until they reach a maximum value, and they bind to decrease. This behavior is related to the change of the electron and impurity atom confinement in the quantum well. It should be noted that the donor binding energy increases with nitrogen concentration. As the nitrogen concentration increases, both the electron effective mass and the band discontinuity increase, while dielectric constant decreases. In this case, the coulombic interaction between the electron and a donor impurity increases, and therefore, the impurity binding energy increases.

**Figure 2:**
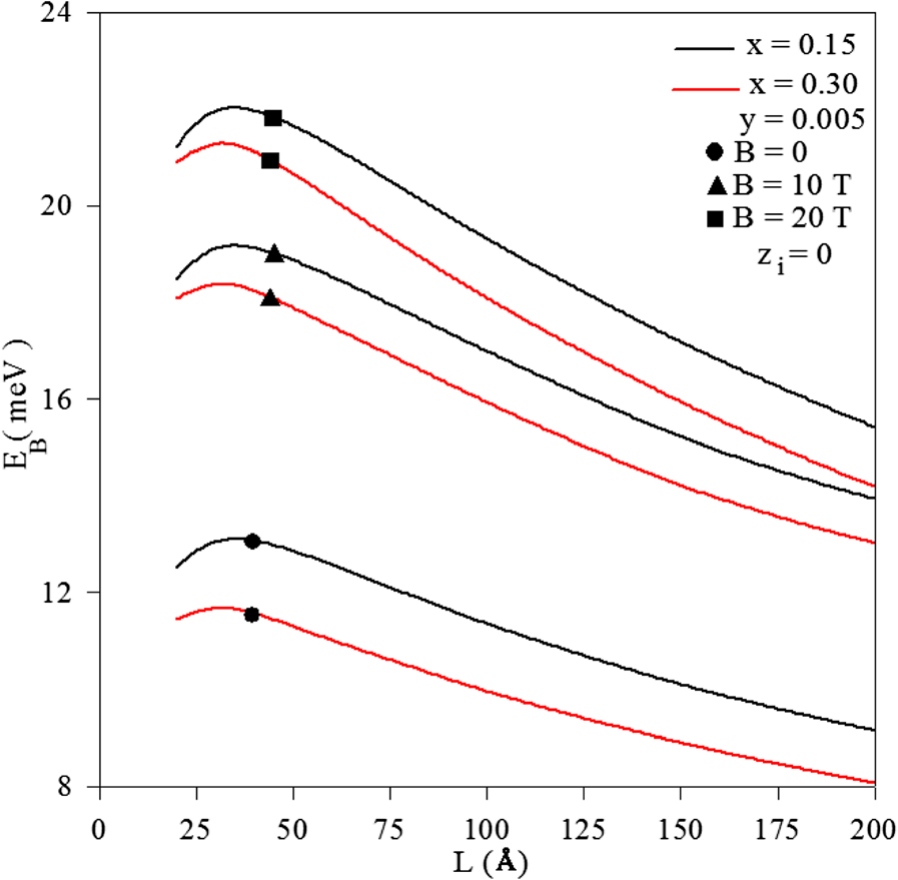
**Variation of impurity binding energy as function of well width**: **magnetic**-**field values and indium concentrations.** This is for different values of the magnetic field and for two different indium concentrations (*x* = 0.15 and *x* = 0.30).

In Figure 2, we have presented the variation of the binding energy for a donor impurity placed on the center of a Ga_1−*x*_In_*x*_N_*y*_As_1−*y*_/GaAs quantum well as a function of the well width for different values of the magnetic field and for two different indium concentrations (× = 0.15 and × = 0.30). It is seen that the impurity binding energy decreases with the well width for the considered values of the indium concentration. This behavior can be explained as follows: the band discontinuity and the dielectric constant increase with the increasing indium concentration; on the other side, the electron effective mass decreases. Thus, by increasing the indium concentration, particles get to be more energetic and they can penetrate into the potential barriers easily where the wave functions of the particles reflect their three-dimensional character, and the probability of finding the electron and hole in the same plane weakens. This behavior weakens the Coulomb interaction between the electron and impurity atom, and the binding energy begins to decrease.

**Figure 3:**
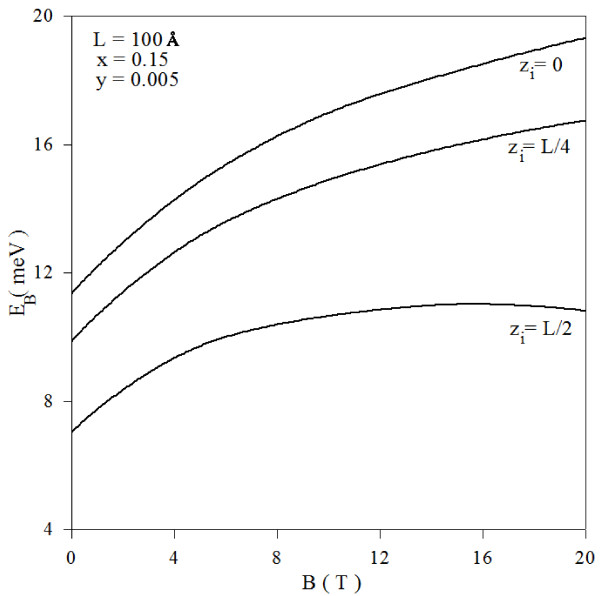
Variation of impurity binding energy as function of magnetic field and for two different impurity positions.

The calculated impurity binding energy for a hydrogenic donor in Ga_1−*x*_In_*x*_N_*y*_As_1−*y*_/GaAs quantum well as a function of the magnetic field (*B*) is given in Figure 3 for different impurity positions (*z*_i_) and well width (*L* =100 Å). As expected, when the magnetic field increases, the binding energy also increases. It can be clearly seen that the impurity binding energy decreases as the position of the impurity approaches the potential barriers. This is because the electron impurity distance increases when the position of the impurity approaches the potential barriers. This leads to the weakening of the electrostatic interaction, therefore to the decreasing in value of impurity binding energy.

## Conclusions

As a summary, we have investigated the effects of the magnetic field, the impurity position, and the nitrogen and indium concentrations on the impurity binding energy in a Ga_1−*x*_In_*x*_N_*y*_As_1−*y*_/GaAs quantum well in this study. The calculations were performed within the effective mass approximation. We have found the impurity binding energy on the magnetic field, the impurity position, and the nitrogen and indium concentrations. This case gives a new degree of freedom in device applications, such as near-infrared electro-absorption modulators and quantum well infrared detectors, and all optical switches. We hope that our results will stimulate further investigations of the related physics as well as device applications of group III nitrides.

## Competing interests

The authors declare that they have no competing interests.

## Authors' contributions

IS and HS defined the theoretical framework of the study. UY, FU, and SS conducted the numerical calculations, prepared computer programs, and gathered the research data. EK, CD, and MMR analyzed the data findings and contributed on conclusions. All authors read and approved the final manuscript.
